# Perceived corporate social responsibility and pro-environmental behaviour: Insights from business schools of Peshawar, Pakistan

**DOI:** 10.3389/fpsyg.2022.948059

**Published:** 2022-07-28

**Authors:** Sana Tariq, Mohammad Sohail Yunis, Shandana Shoaib, Fahad Abdullah, Shah Wali Khan

**Affiliations:** ^1^Sarhad University of Science & Information Technology (SUIT), Peshawar, Pakistan; ^2^Institute of Management Sciences, Peshawar, Pakistan

**Keywords:** pro-environmental behavior, developing country, stakeholder theory, stakeholder salience, corporate social responsibility

## Abstract

Corporate Social Responsibility (CSR) and environmental sustainability have become urgent concerns for contemporary businesses. This study focuses on the interplay between corporate social responsibility perceptions and pro-environmental behaviour (PEB) in response to experts’ call for research on the micro-foundations of corporate social responsibility. In addition, it reveals the mechanism underpinning how perceived CSR shapes pro-environmental behaviour in an understudied developing context. Empirically, a qualitative multiple-case research design is utilised by selecting three business schools from Peshawar, Pakistan. Fourteen semi-structured interviews were conducted with senior management and faculty to collect data. Besides primary data, a qualitative documentary review is used to enhance the research. Data analysis is done through the thematic network technique. Plantation, cleanliness, waste reduction, and energy conservation are the environmental aspects of CSR as regarded by employees. In addition, perceived CSR shapes pro-environmental behaviour via environmental knowledge and awareness, eco-civic sense, environmental values, personality traits, religious perspective, and perceived organisational support for the environment. This study provides original additions to the CSR literature by suggesting eco-civic sensibility and religious perspective as new CSR drivers for pro-environmental conduct. Incorporating stakeholder salience into the context of the present study also advances CSR research. The findings are also valuable for management to make the CSR agenda of business schools more strategic, comprehensive, and centred on the priorities of salient stakeholders.

## Introduction

Concerns about growing socioeconomic and environmental issues around the world have intensified in the modern era. To establish a sustainable society, all sections of society must be accountable in this respect. Organisations recognise the value of their employees and thus encourage them to take part in various activities and contribute to the organisation’s overall performance (Rodrigues da Costa and Maria, 2019). Similarly, most scholars concur that employees are the key to enhancing the environmental performance of their firms (Kim et al., 2019; Anwar et al., 2020). Strategic initiatives cannot be realised without the support and participation of workers (Jenkin et al., 2011; Siddique and Yunis, 2018). It would therefore be imprudent to disregard their strategic importance in environmental performance. For the efficient execution of sustainable initiatives, it is vitally necessary to notice and influence the behaviour of individuals to reduce the environmental footprints of their activities, as well as to give serious consideration to environmental sustainability (Anwar et al., 2020). It is essential to determine the factors of employee participation in sustainable behaviours and its implications for enterprises and the environment (Lamm et al., 2015). Keeping in mind the importance of employees, firms are encouraging their staff to engage in sustainable behaviour and investigating strategies to shape their pro-environmental conduct.

Regarding environmental performance, the higher education industry merits significant consideration. Implementation of the United Nations 2030 Agenda for Sustainable Development, which requires coordinated efforts from all sectors and stakeholders, has increased the significance of engagement by higher education institutions (HEIs). Education is essential to the promotion of a sustainable society (Rodríguez Bolívar et al., 2013). HEIs are the key organisational citizens in promoting sustainable development and social responsibility among future professionals (Gomez et al., 2018). Particularly, business schools operating as organisations that mediate business-society ties might take the initiative in fulfilling their broader social role for the promotion of a sustainable society. In emerging economies such as Pakistan, the social and environmental performance of business schools is significantly more important. This is because social and environmental crises are of a scale significantly bigger than in the industrialised world, and the government’s ineffectiveness lack addressing them. Business schools can contribute to environmental sustainability by fostering eco-friendly employee behaviour.

Corporate social responsibility is considered a novel approach for managing stakeholder relationships and creating value for employees, shareholders, consumers, suppliers, society, and the environment (Manente et al., 2014; Yunis et al., 2017). It provides innovative ways to address social and environmental issues (Dahlsrud, 2008). CSR can handle significant and complicated socioeconomic and environmental issues in an inventive and persistent manner, resulting in long-term outcomes. Organisations recognise the significance of CSR in attaining sustainable development (Moon, 2007) and promoting sustainable behaviours (Murtaza et al., 2021). Since perceptions are an indicator of an individual’s external behaviour and organisational success (Parker et al., 2003; Larsman et al., 2012), it is crucial to comprehend an individual’s CSR perspective on pro-environmental behaviour. Individuals’ impressions of their organisation’s CSR affect their pro-environmental conduct (Celma et al., 2014; Murtaza et al., 2021). Literature is still in its infancy about the processes that explain how and why CSR perception motivates employees to engage in pro-environmental behaviour (Afsar and Umrani, 2020; Murtaza et al., 2021), suggesting that its underlying mechanism is under-explored (Afsar and Umrani, 2020; Shah et al., 2021; Wei et al., 2021).

Unfortunately, CSR research in higher education is sparse (Binsawad, 2020) including in Pakistan. Information regarding employees’ CSR perceptions and behaviours in Pakistan’s HEIs is limited (Asrar-ul-Haq et al., 2016). Particularly, there is a dearth of research on CSR and pro-environmental behaviour in Pakistan’s higher education sector (Jilani et al., 2021). In addition, the stakeholder salience approach is not widely adopted in the higher education industry (Avci et al., 2015; De la Torre et al., 2018). Regarding Pakistan, it is difficult to locate CSR studies that advocate a stakeholder salience perspective in higher education institutions. Only two studies in the existing literature shed insight on employees’ CSR perceptions in the higher education sector (see Asrar-ul-Haq et al., 2016; Ahmad et al., 2017). However, the stakeholder salience lens is not used as the theoretical basis for these analyses. Therefore, CSR research from an environmental management perspective that incorporates stakeholder salience is nearly non-existent in business schools.

Based on the preceding explanation, this study aims to comprehend the employees’ CSR perceptions of pro-environmental behaviour in the business schools of Peshawar, Pakistan. The stakeholder salience viewpoint of Mitchell et al. (1997) informs the current investigation. Therefore, this study has provided an answer to how employees’ perceptions of CSR influence their pro-environmental behaviour, which is vital for the sustainability of an organisation.

### Research context: Pakistan, Peshawar, and business school

#### Pakistan

Pakistan is the sixth most populous country in the world, with a population of over 200 million. Pakistan’s economy is informal and underdeveloped. The regulatory structure of the country is weak. Many issues, including corruption, terrorism, sectarian conflict, poverty, lack of education, healthcare, sanitation, high unemployment, child labour, energy crises, and so on, plague the nation. In addition, the country’s significant environmental challenges include climate change, pollution (air, water, and soil), natural catastrophes, deforestation, and drought caused by the lack of water. Pakistan is ranked 142 on the Environmental Performance Index (EPI) 2020 list of 180 countries. This country’s environmental performance demands aggressive participation from all sectors to prevent a deterioration of the situation and address environmental concerns.

#### Peshawar

The unique geographical backdrop of this study is the city of Peshawar. It is the provincial capital and the largest city in Khyber Pakhtunkhwa (KPK). With almost 2 million inhabitants, it is the sixth-largest city in Pakistan. Most people are Muslims. Overall, the city’s economy is inconsistent and fragile. The Afghan War’s political instability and the influx of migrants have strained the city’s infrastructure. In addition, the magnitude of the displaced population within and surrounding the city has exacerbated its challenges. There are few educational and employment options in the city. Electricity and natural gas shortages have also negatively damaged the city’s economy. Sanitation and pollution are significant concerns. Peshawar ranks among the most polluted cities in the nation. Currently, the city is confronted with precarious environmental conditions. Billion Tree Tsunami is one of the most prominent efforts taken by the provincial government to address air pollution in the city in recent years. This and other ideas should be considered to address the city’s difficulties. The social contribution of other sectors is extremely desirable for enhancing city circumstances.

#### Business school

Among higher educational institutions, business schools are chosen because of their significance and relevance to the issue under inquiry. These institutions provide business education and produce graduates for the corporate sector; hence, they not only provide links between academia and the corporate sector but also facilitate business-society relationships. This highlights that CSR scholars investigating CSR and business-society linkages should examine business schools. In Pakistan, business schools seeking accreditation from the National Business Education Accreditation Council (NBEAC) must fulfil social responsibility (accrediting council for business schools in Pakistan). This suggests that responsible behaviour should be an intrinsic component of the business school’s ideals and strategy, as well as its daily operations. This study is within the setting of three business schools, namely the Institute of Management Studies (IMS), Institute of Management Sciences (IM| Sciences), and Department of Business Administration (DBA) of Sarhad University. The term “school” is used to designate an entity, whether it is a free-standing business school or a faculty, school, or department within a university (NBEAC, 2022). Thus, the selected cases represent the business schools in Peshawar, Pakistan. These schools are selected because of the following reasons: The Institute of Management Studies is affiliated with Peshawar University (the first-ever and oldest public university in the country and the mother institution of the province); Institute of Management Sciences is the premier (public sector-autonomous) business school in the province of KPK and is certified by NBEAC; The Department of Business Administration at Sarhad University (private sector) is one of the province’s few private business schools accredited by NBEAC. In addition, business schools are chosen based on the affiliations of the researchers with each of them. Being directly associated, the researchers know that these institutions practise CSR to varying degrees, and it also facilitated data collection.

## Literature review

Organisations are being compelled to dedicate a large amount of time and resources to address rising social and environmental challenges worldwide. Pollution, ozone layer depletion, climate change, deforestation, wildlife injury, waste management, sanitation, and a lack of natural resources such as clean water, to name a few, have all escalated environmental concerns at all levels. All sectors of society are now required to make positive environmental contributions. Organisations across the globe are taking serious steps in this regard (Murtaza et al., 2021) and are actively exploring new ways of sustainable development. To emphasise the importance of environmental conservation, concepts such as green organisational performance, green human resources management, and green marketing have been introduced. According to current thinking, fostering pro-environmental behaviour in individuals can significantly reduce environmental problems (Islam and Managi, 2019; Balundë et al., 2020). Employees are the company’s key source of innovation since they generate new ideas (Mansoor et al., 2021). As a result, encouraging and involving employees in sustainable innovation would be an effective strategy. This may be the reason that recent literature has highlighted the need for employees to boost their environmental performance (Singh et al., 2019).

Corporate social responsibility is acknowledged as a source of achieving sustainable development. Various definitions of CSR explicitly document it. For instance, Aguinis (2011) defines CSR as “context-specific organisational actions and policies that take into account stakeholders’ expectations and the triple bottom line of economic, social, and environmental performance” (p. 855). After analysing multiple CSR definitions, Dahlsrud (2008) identifies five key CSR dimensions namely economic, social, stakeholder, voluntariness, and environmental that are frequently used to define the concept. Therefore, CSR is regarded as one of the vital phenomena for contemporary organisations from the environment management perspective (Molnár et al., 2021). It is believed that environmental problems can only be eased by micro-CSR (corporate social responsibility at the individual level). It is quite recent that scholars have noted the importance of CSR in shaping employees’ behaviour towards the environment (Murtaza et al., 2021). Micro CSR encourages employees to get engaged in behaviours that can make contributions to a sustainable future (Ahmad et al., 2021). Understanding how and why CSR produces certain outcomes might be aided by concentrating on micro-CSR.

Corporate social responsibility literature that emphasises micro-level issues is emerging (Morgeson et al., 2013; Wang et al., 2016; Chaudhary and Akhouri, 2019), but still, it is an under-examined domain. CSR has been examined largely at the macro level institutions and often ignores micro-level focus (such as employees) (Seivwright and Unsworth, 2016; Wang et al., 2016; Haski-Leventhal et al., 2017; Ahmad et al., 2021). Recently, the focus has shifted from macro- to micro-level highlighting how CSR affects the attitudes and behaviours of employees (Ahmad et al., 2021; Kong et al., 2021). Although the potential of CSR in shaping employees’ discretionary behaviour is noted by many researchers (Hameed et al., 2019), the underlying mechanism in shaping extra-role behaviour, particularly pro-environmental behaviour, is still poorly understood (Afsar and Umrani, 2020; Shah et al., 2020; Wei et al., 2021). Thus, unveiling the underlying mechanism would extend the horizons of CSR research.

Usually, the manufacturing sector is largely responsible for the deterioration of the natural environment; the manufacturing industry must take steps to limit its ecological footprint to maintain a healthy natural environment. Consequently, prior studies have focused mostly on the industrial sector/manufacturing industry (Javeed and Lefen, 2019; Jilani et al., 2021). This does not mean that the service sector is exempt from environmental preservation, as its role is equally important (Murtaza et al., 2021). In this view, higher education is of equal value. The role of the education sector has been altered from merely serving as a medium of education to being susceptible towards society and the environment (Wijaya and Krismiyati, 2014). Higher academic organisations have a significant impact on the environment in terms of energy use, material consumption, trash generation, and transportation use, and hence environmental sustainability is critical for them (Akhtar et al., 2022).

The institutional context in developing countries is unique (Yunis et al., 2017), and the importance of CSR in achieving sustainable development within the context of the higher education sector is obvious. CSR in HEIs is more than just teaching and research; it is about being aware of the impact of their operations on stakeholders, environment, and society at large (Idowu, 2008). This underscores the inclusion of a triple bottom line within the CSR domain of HEIs. Imparting triple bottom line thinking to the students can result in the development of excellent leaders, who can make organisations successful without harming their society, economy, and planet (Gomez and Preciado, 2013; Yunis et al., 2020). In particular, the sustainable performance of business schools is regarded as highly important. Business schools serving as the mediators of business-society relationships (Boyle, 2004) have been criticised for producing profit-minded individuals only. What this means is that there are questions about whether business schools can fulfil their larger purpose of developing responsible leaders who are devoted to solving the world’s environmental and social problems. Business schools must recognise that they are not merely responsible for producing products (students) that help firms generate money; rather, they produce managers who can embrace the concept of sustainability while making profit (Amran et al., 2010). The inadequate and slow environmental performance of HEIs is gaining the attention of researchers in terms of behavioural change of teachers as well as students rather than just focusing on learning and adopting technology (Akhtar et al., 2022). For strategic and academic issues, top management is actively involved, while faculty members are primarily responsible for institutional strategy implementation and are the key players in knowledge transfer to future generations. Thus, studying CSR perceptions of top management and academic staff towards pro-environmental behaviour and exploring the factors shaping such behavioural outcomes in business schools is vital. Understanding their CSR viewpoint on sustainable behaviour is especially helpful to generate better sustainable policies and decisions.

Corporate social responsibility is a context-specific phenomenon (Dahlsrud, 2008; Aguinis and Glavas, 2019; Jamali and Karam, 2018); therefore, contextualised research can offer useful insights. Unfortunately, prior CSR research in developing countries has largely addressed the relationship of CSR with a philanthropic mindset (Inekwe et al., 2021), such as charities and donations, while disregarding other dimensions and outcomes of CSR, such as environmental concerns. Consequently, it would be interesting to explore if emerging countries’ CSR scope incorporates environmental considerations. Even though CSR and pro-environmental behaviour have been investigated in Pakistan (see Jilani et al., 2021; Murtaza et al., 2021; Raza et al., 2021), the country’s higher education sector has been overlooked in this respect (Jilani et al., 2021). In Pakistani HEIs, there is a lack of information about employees’ CSR perceptions and practices (Asrar-ul-Haq et al., 2016), particularly in terms of pro-environmental behaviour. Recently, Jilani et al. (2021) carried out a study concerning CSR perceptions and pro-environmental behaviour in the higher education sector but of students only. As far as employee impressions of pro-environmental behaviour in CSR are concerned, this leaves a gaping hole in the research. The current study examines faculty members’ CSR perceptions towards pro-environmental behaviour within the context of business schools in Peshawar, Pakistan, to address the gap in contemporary CSR literature.

### Stakeholder salience perspective: Theoretical underpinning

This study’s theoretical framework is based on the stakeholder theory. The reason being it is regarded as a state of art theory as it applies to different organisations, industries, and cultures. To put it another way, the stakeholder perspective deals with the following aspects: shareholders (clients/customers), employees, business partners (governments and competitors), local communities, and the natural environment (Harrison and Freeman, 1999: Zhao et al., 2012). The stakeholder theory of Freeman (1984) is centred on the idea that an organisation can perform better and survive longer if it effectively manages its stakeholder relationships.

Mitchell et al.’s (1997) theory of stakeholder identification and salience is a substantial contribution to stakeholder research and is based on the normative assumption of stakeholder theory. Mitchell et al. (1997) define stakeholder salience as “the extent to which managers give precedence to competing stakeholder claims” (p. 854). According to the authors, the three attributes namely power, legitimacy, and urgency can identify important stakeholders. Power refers to the extent of a stakeholder’s influence on the organisation (Mitchell et al., 1997). Concerning legitimacy, Suchman (1995) states that “legitimacy is a generalized perception or assumption that the actions of an entity are desirable, proper, or appropriate within some socially constructed system of norms, values, beliefs, and definitions” (p. 574). Stakeholder legitimacy comes up from a legal title, exchange, contract, legal right, moral right, moral interest, or at-risk status interest in the benefits or harms due to company actions (Mitchell et al., 1997). Urgency is defined by Mitchell et al. (1997) as “the degree to which stakeholder claims call for immediate attention” (p. 867). The authors further propose that three groups of stakeholders are generated by the combination of the aforementioned attributes, namely (a) latent stakeholders who possess merely one attribute, (b) expectant stakeholders having two attributes, and (c) definitive stakeholders holding all three attributes. The stakeholder salience perspective is useful for managers to prioritise their responses towards the needs and demands of their stakeholders. Utilising this very theoretical lens is thus an appropriate choice.

The environment has become a critical stakeholder for any company. In addition, key stakeholders in the higher education sector include management and faculty members (Avci et al., 2015; Jongbloed et al., 2008). The salience perspective allows the examination of how CSR activities and outcomes can be enhanced to reflect local needs (Erdiaw-Kwasie et al., 2017). Furthermore, this approach is not widely adopted in the higher education sector (Avci et al., 2015; De la Torre et al., 2018), particularly in developing countries. Only two studies have been published in the literature on the CSR perceptions of employees in Pakistan’s HEIs. However, these studies do not embrace stakeholder salience perceptive as a theoretical foundation. One study by Asrar-ul-Haq et al. (2016) adopts the theoretical lens of resource dependency and another by Ahmad et al. (2017) espouses social identity theory. Hence, it is interesting to examine employees’ CSR perceptions towards pro-environmental behaviour in business schools of Peshawar, Pakistan, through the lens of stakeholder salience. This inquiry will help in bridging the knowledge gap and will contribute to the theory development.

## Research gap

There is a lack of comprehension of why and how CSR influences individuals (Aguinis and Glavas, 2012). In different research settings, environmental conservation has been studied using external factors, such as reducing wastage of resources, improving the efficiency of existing resources, and so on. Blok et al. (2015) examined the pro-environmental behaviour of university employees using external factors namely leadership support and leadership behaviour using a framework known as planned behaviour theory. In addition, the pro-environmental behaviour of university staff was analysed in terms of the use of computers, heating and lighting systems, printers and copiers, and the recycling of goods with utility value (Cheema et al., 2020). Nonetheless, very few studies have tried to explore environment conservation from factors that are internal to employees, such as CSR perception, which could be a paradigm shift in striving for sustainability. The available evidence suggests that CSR positively affects employees (Glavas, 2016). These kinds of studies imply that fostering environmentally conscious behaviour among employees is a crucial missing piece in sustainability. Before external change can occur, an interior shift must take place. Thus, the gap guidance from literature was the external factor employed for encouraging employees to engage in environmentally friendly behaviours and not their internal motivation that shapes their perceptions and creates positive attitudes and ultimately leads to actions that translate into pro-environmental behaviours. According to Tian and Robertson (2019), employees’ perceived CSR positively affects employees’ pro−environmental behaviour. These authors suggest that there is a lack of knowledge regarding how voluntary measures bring about environmental change. In industrialised nations, many studies on employee pro-environmental behaviour have been conducted, but such research is lacking in developing nations and Pakistan, in particular.

## Methodology

Because this was a qualitative investigation, we used an interpretive framework. The interpretive approach is ideal for business and management research, particularly in the areas of organisational behaviour and human resource management (Saunders et al., 2012). Since case studies are frequently used in qualitative studies (Stake, 1995; Yazan, 2015), this study also employed a qualitative case study strategy. A multi-case design was enthusiastically adopted for its replication logic and ability to reveal commonalities and discrepancies across the cases. It is widely accepted that multiple case study designs produce robust and reliable evidence (Baxter and Jack, 2008; Heale and Twycross, 2018). Cases were narrowed down to three (business schools).

Purposeful sampling was espoused as a sampling technique. Purposeful sampling can be used for theoretical/analytical/logical generalisation rather than statistical generalisation. The current study being qualitative in nature was not looking for statistical generalisation but to draw analytical generalisations about how CSR perception of the management and faculty members will impact their pro-environmental behaviour. The qualitative analysis of the data yielded themes that served as the basis for the analytical generalisation. Using the idea of saturation, the sample size was determined. The sample size was restricted to 14 interviewees because this number provided researchers with in-depth and rich information about the phenomenon under inquiry (sample details provided in Table 2). Management and academic members also provided data for the study because they have greater in-depth knowledge of the issue under examination. In Peshawar, administrative staff members are often ill-informed when it comes to CSR and pro-environmental behaviour. It was thus sensible to collect data only from the management and faculty members, omitting administrative staff employees who were not involved in the management or teaching roles.

**TABLE 1:** Sample details.

S. no.	Business school/case	Codes	Location	No. of respondents	Data source & codes	Interview duration
1.	Institute of Management Studies (IMS)	BS1	Peshawar	4	Managerial Member (MS1)	66 min
					Managerial Member (MZ2)	31 min
					Faculty Member (FM1)	50 min
					Faculty Member (FM2)	29 min
2.	Institute of Management Sciences (IM| Sciences)	BS2	Peshawar	5	Managerial Member (MM1)	29 min
					Managerial Member (MU2)	21 min
					Faculty Member (FS1)	56 min
					Faculty Member (FN2)	34 min
					Faculty Member (FM3)	39 min
3.	Department of Business Administration	BS3	Peshawar	5	Managerial Member (MW1)	33 min
					Faculty Member (FG1)	67 min
					Faculty Member (FK2)	43 min
					Faculty Member (FS3)	38 min
					Faculty Member (FR4)	29 min

Source: Authors.

**TABLE 2:** Thematic analysis of selected business schools.

Initial codes	Basic themes	Organizing themes	Global theme
Green Environment	Environmental Awareness and Knowledge	Plantation and Cleanliness	CSR perceptions towards pro-environmental behaviour
Growing Trees			
Greening of Surroundings			
Reducing Air Pollution			
Global warming			
Deforestation			
Climate change			
Volunteer work	Eco-Civic Sense		
Behaviour moulding			
Cleaning places			
Grooming the mentality			
Removing trash			
Teaching the concept of Recycling			
Avoiding wastage of material			
**Switching off lights**			
Spiritual satisfaction	Religious Perspective		
Told by religion			
Religious point of view			
Pious act			
Religious aspect			
Encouragement/appreciations from institute side	Perceived Organizational Support for Environment		
Environmental orientation of management			
Organizations and donors supporting sustainable practices			
Approvals and permissions			
Logistics support			
Financial contribution by institution			
Offering institution facilities for green initiatives			
Self-motivation	Environmental Values	Waste Reduction and Energy Conservation	
Personal nature			
Sensitive towards environment			
Personal priority			
Love for nature			
Connection to nature			
Conscientiousness	Personality Traits		
Considerate			
Open to ideas			
Compassionate			
Thoughtful			
Extrovert			

Source: Authors.

For data collection, a multi-method approach was adopted. Face-to-face semi-structured interviews were used to collect primary data. From November 2020 to April 2021, the interviews took place over 6 months. Every question posed was open-ended, direct, and of a neutral tonality. For the sake of brevity, double-barreled questions were not asked. Interviews were conducted in English because all the participants (top management and faculty members of business schools) were fluent in the language. However, participants were allowed to speak in any language they liked (Urdu or English). All the interviews were audio-recorded after taking permission from the respondents and transcribed verbatim. Documentary analysis (examination of websites and prospectuses of the respective cases) was utilised as the secondary data collection tool. For analysis, Attride-Stirling’s (2001) thematic network technique was adopted. To build a thematic network, basic themes offer starting points and then move inwards towards a global theme. Merriam (1998) suggests that for multiple case studies, the with-case analysis should be followed by cross-case analysis. Thus, as a multiple-case study, both within and cross-case analyses were carried out. With-case analysis utilised quotes from interviews relevant to CSR conception and separate thematic networks were also made in each case. For cross-case analysis, similarities and differences were highlighted across the cases and displayed in tabular form as well.

## Results and discussion

Table 3 presents themes that surfaced from the data were taken into consideration when analysing the themes that were chosen. Thematic analysis is performed with the help of a technique known as thematic network analysis, which shows themes on three different levels, including basic themes, organising themes, and global themes. Besides that, the empirical data are provided as table displays and thematic network diagrams.

**TABLE 3:** Themes emerged from raw data of interviews.

Themes	Extracts from interviews
Environmental Knowledge and Awareness	“I would like to create awareness amongst people to keep the places clean” (MU2-BS2).
	“Our students, some of them have started their own businesses, some will join some businesses. If they learn these socially and environmentally responsible knowledge and techniques, they are developed in such a way that they care about the social issues, environmental issues, climatic issues, so whenever they are in good positions, they will contribute more than what they have contributed here, they will be effective movers and they will be champions of those causes and that I think so is very important and relevant for us” (MM1-BS2).
	“The environment in which we live, if we are damaging it and damaging it and damaging it and not doing anything about it, then that is bad” (FM2-BS1).
	“If I consider corporate social responsibility, the problems, issues that arise, pollution or anything, environment, eco system all these come in it” (MS1-BS1).
Eco-Civic Sense	“In our society that civic sense is lacking…I don’t know how many times I have said this, but I focus a lot on it so that I should not see garbage in the class. You have seen our campus; it is very clean campus. So, there is no concept that you must throw trash” (FM3-BS2).
	“I am sure if our students have heard me or someone else so many times that use less water, don’t waste water, so when they go out, they will use this” (MM-BS21).
	“Once one of my students told me, after he had graduated, that he was going somewhere and, on the road, he saw some broken glass or can or paper or something. He picked it up and put it in a proper place. So, you know this is rewarding for me that a student has learnt something from me, other than the academic side” (MM1-BS2).
Religious Perspective	“Our prophet Muhammad (PBUH) said that cleanliness is half the faith” (FS1-BS2).
	“There are many instructions in Islam about cleanliness, so keeping ourselves and surroundings, roads, parks etc. clean is important to us from religious point of view too” (FM1-BS1).
	“Our religion tells us to clean ourselves and to keep our surroundings clean so definitely we will move towards that” (MW1-BS3).
	“Planting trees is charity in our religion” (FM3-BS2).
Environmental Values	“The environment in which I am living, that matters the most to me, and it is the one thing which motivates me, which implies me to act in that pro-environmental manner” (FS3-BS3).
	“I try not to print a lot. I try not to waste a lot of papers because I think about that we are wasting papers. We must take care of these things” (FM1-BS1).
	“If there is realization in an individual himself/herself so will give importance to such initiatives, will take on it and would like to have related things to be done” (FM3-BS2).
	“I try to utilise resources in the best way, so that they should not be wasted. Summer is around but I haven’t started using air conditioner at office till now” (MS1-BS1).
	“When I leave the office, I make sure that I have switched off all the lights” (FS1-BS2).
Personality Traits	“I am basically an environmentalist, so I think from that perspective’. And being a visionary person, I see that we need to protect our environment more than protecting our borders. If we want the world to be lived by humans for a long time, we need to work on earth and the businesses; we can play a very big role in that” (FM1-BS1).
	“I am self-compelled and conscious, so I keep on pushing things” (FS1-BS2).
	“It depends on the individual in a sense that some people do it because they are compassionate” (MZ2-BS2).
Perceived Organizational Support for Environment	“I forgot the year, but we conducted a clean Peshawar activity. Faculty and students went out and cleaned the garbage and collected plastic bags from different areas” (FS3-BS3).
	“Our department has taken many initiatives like green Pakhtunkhwa. We have contributed more than three thousand plants in the society” (FK2-BS3).
	“We have got a ‘Sheenwatan’, which is responsible for tree plantation. So far, we have been able to manage thousands of trees” (MU2-BS2).
	“A teacher is heading our green tree plantation area activities and we are taking donations for getting plants and giving them to schools and colleges in far off areas” (MM1-BS2).
	“It is up to business schools, they should take initiative and should involve their management, faculty, and students in different social issues, e.g., we are facing a lot of pollution, you see a lot of garbage around. They should come up with ideas on how to cope up with these problems. Water reservation or water management is a major issue in Pakistan, so they can encourage their students to go even door to door and try to educate people” (FS3-BS3).
	“Last year IM | Volunteer team did the board bazar cleanliness campaign which PDA, WSSP, Municipal Corporation could not do. We showed them what a bunch of twenty-five or so volunteers can do in just three days in hardly a hundred and thirty-five thousand rupees” (FS1-BS2).

Source: Authors.

The current study provides management and faculty CSR perspectives toward pro-environmental behaviour within the framework of business schools in Pakistan, particularly in Peshawar. Findings suggest the inclusion of environmental dimension within the CSR scope of all the selected business schools. Plantation, cleanliness, waste reduction, and energy conservation represent the environmental domain of their perceived CSR. In addition, the findings of this investigation imply that a micro-CSR perspective moulds environmentally conscious behaviour and reveals the mechanism that lies beneath it. Environmental knowledge and awareness, environmental values, personality traits, eco-civic sense, religious perspective (individual-level factors), and perceived organisational support for the environment (organisational-level factor) are some of the common themes that emerged from the data. These are the CSR-driven factors that contribute to the development of environmentally conscious behaviours in individuals. The management and faculty members of selected cases engage in environmentally friendly behaviours such as planting trees, taking part in cleaning programmes, turning off lights when they are not required, avoiding excessive printing, and so on.

Most of the respondents’ primary worry relates to plantations rather than any of the other myriad environmental problems. Each of the selected cases has managerial and teaching staff that are actively taking part in this endeavour. Nevertheless, IM| Sciences is in the lead and the activity in question is carried out methodically. There is a society at this business school known as “Sheenwatan” that is committed to this one activity, which is tree planting. On the grounds of the school, there is a specific location set aside to cultivate and tend plants. The planting of thousands of trees in Peshawar and the surrounding areas has been made possible via this platform. In addition, the idea of a sustainable and green campus is practised at IM| Sciences. Both the IMS and SUIT business schools have a lot of room for improvement in this regard. Cleanliness is the second prioritised area of management and faculty members of the selected business schools. However, the scope is primarily limited to ensuring that the campus and its surrounding areas are clean. In addition, the findings of this research show that reducing trash and conserving energy are also examples of environmentally friendly behaviours, albeit ones that were detected to a lower level. These and other connected topics, like recycling, conserving water, and using fuel-efficient transportation choices require attention.

**FIGURE 1:**
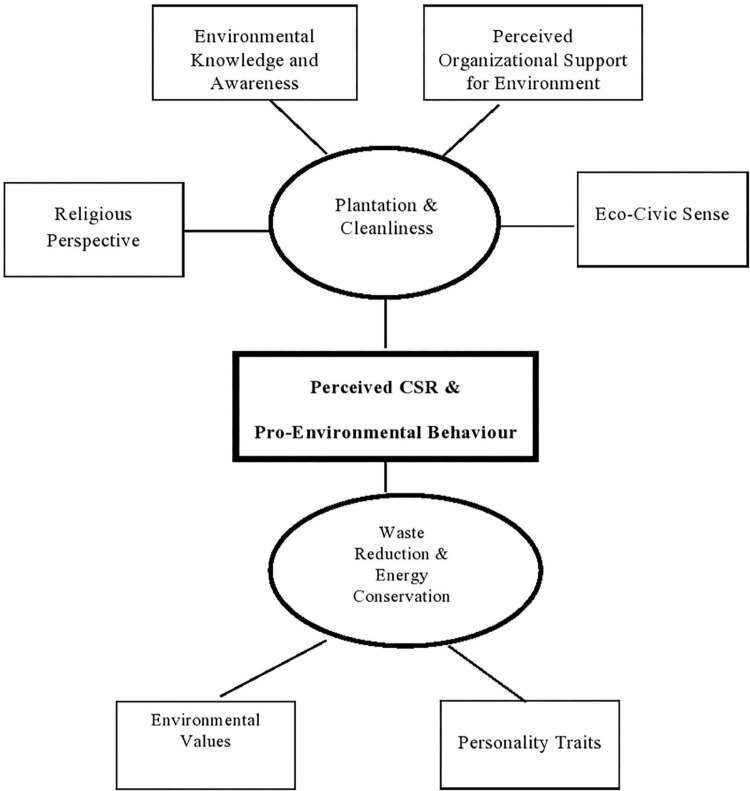
Thematic network diagram of perceived CSR and pro-environmental behaviour in business schools of Peshawar, Pakistan.

Empirical data reveal, in a nutshell, that the management and teaching staff of all the selected business schools regard environmental issues as part of their CSR efforts and are fairly interested in green and clean projects. This is the conclusion that can be drawn from the data. Nevertheless, participation in these activities is entirely voluntary; they are also less formal and have a more constrained scope. Table 1 summarises the thematic analysis conducted on a selection of business schools, whereas Table 2 outlines themes that arose from the data. A diagram of thematic network connections is presented in Figure 1.

Empirical evidence suggests that the micro CSR perspective within the context of business schools in Peshawar, Pakistan, incorporates environmental orientation. The inclusion of environmental perspective sheds light on the fact that the CSR domain of developing countries is expanding. This can be attributed to complex and vulnerable environmental conditions in Pakistan. So, besides government programmes like “Billion Tree Tsunami” and the “Clean Green Pakistan Movement,” the local community expects diverse sectors to contribute to this effort. Thus, both management and faculty of selected business schools believe that they are equally accountable and responsible for improving the environment. Integration of the environmental dimension of CSR within the strategic agenda of business schools can give exposure to new responsibilities, new habits, new values, and new knowledge, ultimately leading to sustainable innovations.

Environmental concerns are a social obligation, as evidenced by empirical data, which show that respondents are well-versed in the country’s environmental challenges and problems. This translates into their pro-environmental behaviours. A person’s pro-environmental behaviour is defined as a set of behaviours that safeguard the natural environment from the harm that human activities cause it (Kim et al., 2015). Employee pro−environmental behaviour is a workplace−specific environmental behaviour (Norton et al., 2015). Literature supports the findings. It is unlikely that an individual will be intentionally concerned towards the environment or act deliberately in a pro-environmental manner if that individual has no knowledge about the problem or prospective positive actions (Gifford and Nilsson, 2014). When organisations engaged in CSR share their CSR practices through newsletters, e-mails, seminars, and so on, their employees gain awareness and knowledge of the existing social and environmental concerns in which their organisations are participating (Arnaud and Sekerka, 2010; Rafiq et al., 2016). An individual’s environmental knowledge and awareness can lead to an individual’s concern for efficiently reducing ecological issues and promoting environmentally friendly behaviour in an organisation (Renwick et al., 2013; Baumgartner and Winter, 2014; Fawehinmi et al., 2020). Furthermore, findings reveal that environmental values make an individual intrinsically motivated to display sustainable behaviour. All the selected cases are supported by the literature. CSR promotes a culture in which personal environmental values flourish and lead to pro-environmental behaviour in employees (Zientara and Zamojska, 2016). An individual’s perception that his/her organisation is responsive to society and the environment would translate into pro-environmental behaviour only when an individual’s moral schemas, moral motives, and moral values guide him or her to get involved in positive behaviours (Afsar and Umrani, 2020). Similarly, empirical evidence suggests that respondents’ personality traits influenced by CSR are positively associated with their pro-environmental behaviour. Our personalities are profoundly shaped by the circumstances in which we live. A combination of genetics and our environment shapes human personalities, according to research. In other words, it proves that personality traits are something that can be cultivated. Hence, a CSR supportive environment drives the personality dimensions of its employees making them behave in a more socially and environmentally responsible manner. There is a good correlation between this study and previous ones (see Fraj and Martinez, 2006; Swami et al., 2011; Markowitz et al., 2012; Yunis et al., 2017).

In addition, empirical evidence shows that management and faculty pro-environmental behaviour result from perceived organisational support for environmental sustainability. Perceived organisational support towards the environment motivates employees to reciprocate it through their environmental performance. Literature also supports this conclusion (see Lamm et al., 2015; Wesselink et al., 2017). Ramus and Steger (2000) also assert that employees’ positive perceptions about their organisational commitment to pursue environmental protection make them more likely to generate ideas for improving the environmental policies of the company. This suggests that if employees perceive that their organisation CSR includes environmental concerns, they will explore new and better ways to protect the environment, leading to sustainable innovations.

It also becomes evident from data that serious problems such as polluted places, unhygienic environment, and water scarcity to name a few are due to the lack of civic sense among residents of the country. Data suggest that the promotion of civics sense falls within the CSR domain of selected cases and positively contributes to shaping sustainable behaviour. Employees who have a keen sense of civic duty are more likely to engage in environmentally friendly practices. When it comes to Pakistan, there is a lack of civic awareness. Most people either do not know about it or have little interest in doing so. It is an excellent opportunity for business schools to come up in this area and promote civic sense in their employees, which can further be imparted to their graduates. It would improve the sustainable orientation of the business schools as well as future organisations. Hence, this research provides a unique insight into how cultivating a strong civic sense contributes to long-term sustainability.

The basic essence of CSR has common ties with the religion of the country, which is Islam. Many scholars propose that environmental concern is embedded in religious beliefs and values (see, for example, Gifford and Nilsson, 2014; Anderson et al., 2018). Therefore, religious perspective is also emphasised by participants in all of the cases to participate in social and environmental initiatives. Plantation and cleanliness are the most important environmental responsibilities of business school managers and teachers, according to empirical evidence. Cleanliness is among the basic tenets of Islam and tree plantation is regarded as “Sadqa-e-Jariyah” (continuous charity). These constituents turned out to be the major driver of the pro-environmental behaviour of the respondents. Pashtun/Pathan people make up the bulk of the population in Peshawar, Pakistan. Pashtuns are known for their deep devotion to and affiliation with religious ideas, thus religion is intrinsic to them. For this reason, it should come as no surprise that religious views guide Pashtuns’ participation in sustainable efforts. Hence, the current study’s discovery of how religious perspective shapes pro-environmental behaviour is fresh and intriguing.

In addition, the stakeholder salience lens helps to comprehend the CSR perspective of employees towards pro-environmental behaviour. Due to the deteriorating environmental circumstances around the world, the environment has become a salient stakeholder for every type of organisation. The time is ripe for action on environmental issues. Hence, the environmental perspective is included in the micro-CSR perspective because of its saliency. It is also evident from the extant literature that demands powerful and legitimate stakeholders get a quick response from the firms (Parent and Deephouse, 2007; Currie et al., 2009; Yunis, 2012). An organisation’s long-term sustainability can be ensured by meeting the demands of its constituents (Jamali, 2008). Stakeholder salience is also said to influence CSR practices and preferences (Brammer and Millington, 2003; Alniacik et al., 2011).

Summing up, the findings of this study show that the micro-CSR perspective of business schools in Peshawar includes an environmental dimension. CSR-pro-environmental behaviour association is also examined in this study, which reveals the underlying mechanism of the relationship. The results are unique because they provide novel factors such as eco-civic sense and religious viewpoints that shape the pro-environmental orientation of management and teaching staff of business schools operating in Peshawar, Pakistan. It also offers a new viewpoint by theorising CSR and pro-environmental behaviour through the lens of stakeholder salience, which opens new research paths. Stakeholder salience theory explicitly addresses the relationship between stakeholder attributes, which include their activities. Stakeholder salience is a valuable addition to the traditional stakeholder theory, in that it takes into account the practical reality in which the management considers the stakeholder’s claim for two reasons: first, the management feels it is the right thing to do, and second, to achieve organisational goals (Mitchell et al., 1997).

Thus, stakeholder salience posits that employees are the most important stakeholder when it comes to upholding organisational CSR attempts and influencing their behaviours in accordance with the sustainable policies of the organisation (McWilliams et al., 2006; Ardito and Dangelico, 2018). Harvey and Schaefer (2001) focusing on the environmental issues claim that the employees have significant salience when it comes to environmental protection. Because environmental disclosure is a component of CSR disclosure, employees’ perception of CSR will impact their pro-environmental behaviour. Employees tend to associate themselves with groups to which they belong and they try to achieve the group’s goals with which they associate (Park and Levy, 2014). When organisations indulge in CSR practices, their employees identify more strongly with such activities because they feel part of this bigger group and it enhances their self-esteem (Farooq et al., 2017). Pfarrer (2010) suggests CSR initiatives increase organisational performance through the stakeholder’s theory of CSR as CSR is the company’s stakeholders’ responsibility. CSR policies of a corporation are viewed as beneficial to the company’s workers and the environment. Employees’ perception of CSR has become more relevant in today’s context where environmental problems have created innumerable challenges for its sustainability. It is thus imperative for organisations to adopt socially responsible policies to gain the stakeholders’ trust.

## Conclusions and implications

The employees of an organisation are the organisation’s most important stakeholders. Their viewpoints significantly influence their attitudes and behaviours in a variety of settings. The same idea applies to environmental performance: the greater their support and engagement, the greater the likelihood of success. CSR programmes assist businesses in establishing a culture that seeks to cultivate the environmental attitudes, norms, and values of individual employees to urge them to engage in environmentally conscious actions. The dedication of employees to promote environmentally friendly ideas and aid in implementing environmental regulations are crucial elements of the corporate social responsibility activities that organisations engage in. The pro-environmental conduct of employees is crucial for an organisation to achieve sustainable environmental development, which is required for the effective implementation of environmental protection strategies.

The need of the hour is for employees to act in environmentally responsible ways. Employees are required to act in socially responsible manner due to increased environmental concerns and worldwide efforts to reduce global warming. If sustainable projects are to be properly implemented, it is necessary to recognise and change the behaviours of individuals to decrease the environmental footprints that their activities leave behind and to place a major emphasis on environmental sustainability. This is necessary for the effective execution of sustainable programmes. Therefore, businesses are actively seeking ways to promote environmentally sustainable practices by encouraging employees to perform in a manner that is good for the environment. The findings of this study are unusual in the sense that they identify two new factors that shape the pro-environmental behaviours of the management and teaching staff of business schools operating in Peshawar, Pakistan: eco-civic sense and religious perspective. This distinguishes the findings of the present investigation. Moreover, the findings of this study offer a novel perspective by reexamining CSR and pro-environmental behaviour via the lens of stakeholder salience. In addition, these discoveries open new study avenues for future investigation. Embracing stakeholder salience in the context of the current study also takes CSR research a step further. The findings are also beneficial for management in making the CSR agenda of business schools more strategic, comprehensive, and centred on the priorities of salient stakeholders.

This study contributes to the subject of corporate social responsibility first by empirically demonstrating a micro-CSR approach to environmentally responsible behaviour in the setting of business schools in Peshawar, Pakistan. Since manufacturing has historically been the primary focus of research in this field, corporate social responsibility and environmental orientation are rarely explored in the context of the service sector (Javeed and Lefen, 2019). Higher education institutions in the country are particularly understudied in this regard (Jilani et al., 2021). Hence, this is a novel piece of study that provides a fertile environment for CSR research. Second, considering the context-specific nature of CSR (e.g., Dahlsrud, 2008; Yunis, 2009; Aguinis and Glavas, 2019; Jamali and Karam, 2018; Khan and Yunis, 2019), the adoption of a developing country perspective (Pakistan) is a significant contribution to the understanding of local or context-specific orientation. Third, this study adds to the existing body of knowledge by throwing light on the underlying mechanism and exploring the factors that shape the extra-role behaviour of employees, specifically pro-environmentally behaviour. Both eco-civic awareness and religious outlook emerged as distinguishing characteristics. Fourth, the utilisation of a stakeholder salience framework is a significant theoretical contribution to this investigation. When Pakistani higher education institutions are considered, it may be difficult to locate studies that advocate for stakeholder salience orientation and CSR. Therefore, this is an exceptional novel study, as it combines stakeholder salience with the perspective of micro-CSR on pro-environmental behaviour in Peshawar, Pakistan’s business schools. Fifth, the findings of the present study may assist senior management in making the CSR agenda of business schools more strategic, comprehensive, formal, and stakeholder-focused. It would be beneficial for policymakers of higher education institutions to raise their understanding of corporate social responsibility and to address environmental issues by encouraging sustainable employee behaviour.

### Limitations and future direction

This study contains limitations, as any do. The research is limited to business schools in Peshawar, Pakistan, to understand employees’ CSR opinions on pro-environmental behaviour. Another problem is purposeful sampling. Purposeful sampling is prone to researcher bias. Subjective, non-probability-based unit selection makes sample representativeness difficult to argue. This further undermines the researchers’ theoretical/analytical/logical generalisations. Future research should compare business schools in other Pakistani cities and developing countries.

This study collected data from a few schools in the provincial capital. Future research could include student opinions. Demographic attributes, such as age, gender, education level, and so on, of internal stakeholders are ignored. We wonder if younger and older employees’ CSR perspective shapes their pro-environmental behaviour in the same or diverse ways. Younger generations contribute more to environmental efforts than older generations do. Future CSR research should include stakeholder demography. Such studies could advance CSR knowledge. Future research could use the study’s theoretical foundations (stakeholder salience) to confirm the findings in Pakistani and other developing environments. Moreover, this work uses qualitative research. Small sample sizes restrict qualitative studies’ (external validity) generalisability (Lent et al., 2019). Future research could use a large sample size and quantitative methodology to validate and generalise these findings for Pakistan and other developing nations. Cross-sectional studies allow for future longitudinal CSR research.

## Data availability statement

The original contributions presented in this study are included in the article/supplementary material, further inquiries can be directed to the corresponding author/s.

## Author contributions

ST: main idea and key contributor, write-up. MY: framing and theoretical insight. SS: formatting, references, and editing. FA: data collection and arrangement, thematic analysis. SK: formatting, data collection, and analysis. All authors contributed to the article and approved the submitted version.
